# Maturity-Onset Diabetes of the Young 10 (MODY10): A Comprehensive Review of Genetics, Clinical Features, and Therapeutic Advances

**DOI:** 10.3390/ijms26168110

**Published:** 2025-08-21

**Authors:** Ali Mazloum, Sofya G. Feoktistova, Anna Gubaeva, Almaqdad Alsalloum, Olga N. Mityaeva, Alexander Kim, Natalia A. Bodunova, Mary V. Woroncow, Pavel Yu Volchkov

**Affiliations:** 1 Federal Research Center for Innovator and Emerging Biomedical and Pharmaceutical Technologies, Moscow 125315, Russia; feoktistova_sg@academpharm.ru (S.G.F.); alsallum_a@academpharm.ru (A.A.); mityaeva_on@academpharm.ru (O.N.M.); volchkov_py@academpharm.ru (P.Y.V.); 2 Moscow Clinical Scientific Center N.A. A.S. Loginov, Novogireevskaya Street, Moscow 111123, Russia; n.bodunova@mknc.ru; 3 Faculty of Fundamental Medicine, Lomonosov Moscow State University, Moscow 119991, Russia; mworon@inbox.ru

**Keywords:** Type 1 diabetes, monogenic diabetes, MODY10, gene therapy

## Abstract

Maturity-onset diabetes of the young type 10 (MODY10) is a monogenic diabetes subtype caused by heterozygous mutations in the insulin gene (*INS*), leading to defective proinsulin processing, endoplasmic reticulum (ER) stress, and β-cell dysfunction. Current management relies on sulfonylureas or insulin therapy, but these fail to address the underlying genetic defect. Recent research has elucidated the molecular mechanisms of MODY10, including ER stress induced by proinsulin misfolding, activation of the unfolded protein response (UPR), and β-cell apoptosis. Emerging therapies such as Adeno-Associated Virus (AAV)-mediated gene delivery to induce the glucose-responsive hepatic insulin expression, plasmid-based single-chain insulin analogs, and cell-based therapies show promise in preclinical studies. However, critical challenges remain, including immune responses to AAV vectors, incomplete correction of dominant-negative mutant effects, and the need for long-term safety data. This review summarizes current knowledge on MODY10 genetics, pathophysiology, and therapeutic innovations, while identifying key gaps for future research to enable precision medicine approaches.

## 1. Introduction

Diabetes mellitus (DM) is a chronic metabolic disorder characterized by persistent hyperglycemia resulting from defects in insulin secretion and/or insulin inefficiency [1]. According to the International Diabetes Federation, in 2024, over 589 million adults worldwide were diagnosed with diabetes, and the numbers are estimated to increase based on an annual growth rate of 3.2%, reaching 853 million cases by 2050 [2]. The disease is classified into several subtypes, with the most prevalent being type 2 diabetes (T2D), accounting for 90–95% of all cases. Type 1 diabetes (T1D) contributes to between 5% and 10%, while other rare forms account for 1–3% of the global cases. Diabetes subtypes differ in etiology, pathophysiology, and management.

Type 1 diabetes is an autoimmune disorder in which the immune system selectively destroys pancreatic β-cells, leading to absolute insulin deficiency [3]. While it typically manifests in childhood or adolescence, it can also develop in adulthood (latent autoimmune diabetes of adulthood—LADA), requiring lifelong insulin therapy and monitoring of lifestyle and diet [4]. In contrast, T2D is the result of a combination of cellular resistance to insulin and progressive β-cell dysfunction, and while often associated with obesity and lifestyle factors in middle-aged adults, it is increasingly diagnosed in younger populations due to rising obesity rates and sedentary behaviors [5]. While these two forms dominate clinical practice, monogenic diabetes syndromes, such as maturity-onset diabetes of the young (MODY), present a distinct and often misdiagnosed subset of diabetes cases.

MODY represents a heterogeneous group of monogenic diabetes disorders that are inherited in an autosomal dominant pattern, where a single copy of the mutated gene from either parent is sufficient to cause the disease [6]. First described in 1974 by Tattersall and Fajans, MODY is characterized by its early onset, typically before the age of 25, although later presentations could occur. Additional characteristics of MODY include primary β-cell dysfunction, absence of autoimmune antibodies and a family history of diabetes in multiple generations, with a 50% chance of offspring inheriting the disease if one of the parents is diagnosed with MODY [7].

Despite advances in genetic testing, MODY remains underdiagnosed, with many cases being misclassified as T1D or T2D, leading to inappropriate treatment [8]. Among monogenic forms, MODY10, caused by *INS* mutations, exemplifies how single-gene defects disrupt insulin biosynthesis, offering unique insights for targeted therapies.

This review sheds light on current knowledge on MODY, emphasizing MODY10, including its genetic basis, molecular mechanism, and emerging approaches, including adeno-associated virus (AAV) gene therapy, to treat it.

## 2. Literature Search Methodology

The source of material used in this review is peer-reviewed articles from PubMed, Google Scholar, and Web of Science (2000–2025) using keywords: “MODY10,” “INS mutations,” “proinsulin processing,” “ER stress in β-cells,” “AAV gene therapy diabetes,” and “stem cell-derived β-cells.”

Inclusion criteria prioritized: Original research on MODY10 genetics/pathophysiology. Preclinical/clinical studies of gene/cell therapies for monogenic diabetes. Reviews/meta-analyses (2010–2025) to contextualize mechanistic insights.

Excluded: Non-English articles, non-peer-reviewed sources, and studies lacking molecular/clinical relevance.

## 3. Genetic Basis of MODY—MODY10

Until recently, distinguishing between childhood-onset diabetes in non-obese patients was challenging. However, with advances in molecular biology techniques and sequencing, the single gene defects causing such diseases have been identified [9]. Monogenic diabetes is the result of mutations in a single gene encoding the potassium channels and glucose-sensor glucokinase in cells, as well as transcription factors or insulin [10].

To date, fourteen MODY subtypes have been identified, each linked with mutations in specific genes (22 genes total) (Table 1).

**Figure 1:**
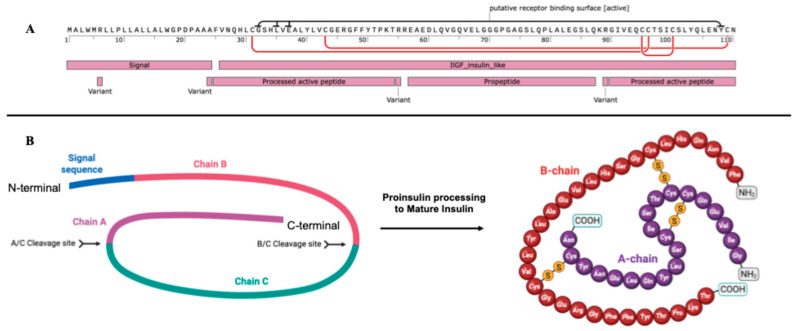
Structure of proinsulin and mature insulin. (**A**) The full coding amino acid sequence of human insulin showing all its features; (**B**) Structure of proinsulin and mature insulin showing the peptide chains, two disulfide bonds between the B-chain and the A-chain, and one disulfide bond of the A-chain. Created in Biorender.

Mutations in a single gene are not always consistent. Different mutations within a gene can lead to truncation in the gene’s coding sequence or misfolding of the protein, potentially causing MODY. For instance, over 20 unique heterozygous mutations in the insulin gene (*INS*) (Chr11p15.5) have been identified, all resulting in MODY10. This gene encodes preproinsulin, the precursor of mature insulin (Figure 1) [12].

**Figure 2:**
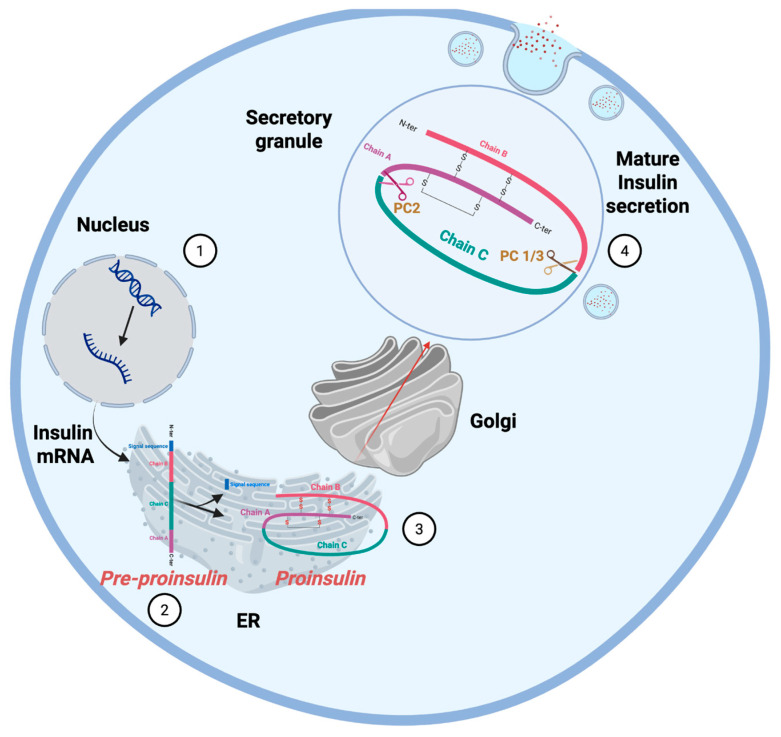
Insulin biosynthesis pathway: From mRNA translation to mature insulin secretion. Created in Biorender.

The synthesis of mature insulin begins with the transcription of the *INS* gene, producing preproinsulin mRNA that is translated by ribosomes on the rough endoplasmic reticulum (ER) into preproinsulin, a 110-amino acid precursor containing an N-terminal signal peptide (24 aa), B chain (30 aa), C-peptide (35 aa), and A chain (21 aa) (Figure 1 and Figure 2, steps 1 and 2) [12].

Preproinsulin enters the ER lumen, where proinsulin (86 amino acids) is formed by cleavage of the signal peptide. Proinsulin then undergoes proper folding facilitated by chaperones such as BiP and PDIA1 (Protein Disulfide Isomerase A1), while forming three critical disulfide bonds (A6–A11, A7–B7, and A20–B19) to stabilize its structure (Figure 2, steps 2 and 3).

Correctly folded proinsulin is transported to the Golgi apparatus via COPII-coated vesicles. In the Golgi apparatus, proinsulin is sorted, concentrated, and packaged into clathrin-coated immature secretory granules that bud from the trans-Golgi network.

Within the granules, proinsulin is cleaved by prohormone convertases PC1/3 (at Arg31-Arg32, separating the B chain from the C-peptide) and PC2 (at Lys64-Arg65, separating the C-peptide from the A chain). Carboxypeptidase E then trims the exposed basic residues to yield mature insulin (51 aa, comprising the A and B chains linked by disulfides) and the C-peptide. Later, both mature insulin and C-peptide are secreted in equimolar amounts (Figure 2, step 4). The mature insulin is stored in secretory granules with Zn^2+^ crystals until glucose stimulation triggers Ca^2+^-dependent exocytosis, releasing insulin into the bloodstream to regulate glucose uptake, while the Golgi apparatus additionally modifies other secretory proteins through glycosylation, sorts cellular cargo, synthesizes lipids like sphingomyelin, and maintains membrane integrity for granule formation [12].

Under normal physiological conditions, up to 10–15% of newly synthesized proinsulin forms mis-paired disulfide bonds due to imperfect oxidative folding [13,14]. This misfolding escalates when β-cells are stressed—either by increased proinsulin demand (e.g., insulin resistance) or a defective ER machinery [15,16,17]. In MODY10, *INS* gene mutations exacerbate this dysfunction by producing proinsulin mutants that misfold and form aberrant disulfide-linked complexes, trapping wild-type (WT) proinsulin and impairing insulin production.

The genotype–phenotype correlation in MODY10 is highly structured: Signal peptide mutations (e.g., c.3G>T) disrupt ER translocation, typically causing neonatal diabetes, and B-chain mutations (e.g., R6C) act in a dominant-negative manner, blocking cleavage by prohormone convertases (PC1/3). Meanwhile, disulfide bond disruptors (e.g., C43G) induce ER stress via misfolded proinsulin aggregation.

To reveal the mechanistic insights of these mutations, researchers like Wang et al. perform structural studies [18]. Their results showed that R6C distorts the B-chain N-terminus, while C43G destabilizes the A7-B7 disulfide bond, generating aggregation-prone intermediates [18]. Despite these clear patterns, phenotypic variability exists—15% of mutation carriers remain normoglycemic, likely due to compensatory mechanisms (e.g., Endoplasmic Reticulum-associated Degradation (ERAD) upregulation or modifier genes like WFS1), and the absence of ketoacidosis suggests residual insulin secretion from extra-pancreatic sources.

Wang, H. et al. further dissected this relationship by characterizing two MODY10-associated mutants, G(B20)R (p.Gly44Arg) and P(B28)L (p.Pro52Leu) [18]. Both impair ER oxidative folding, cause trafficking defects, and trigger apoptosis, but with differing severity: G(B20)R and P(B28)L exhibit partial dominant-negative effects, allowing some WT-proinsulin processing. In contrast, R6C and C43G nearly abolish insulin production, correlating with earlier and more severe diabetes.

Thus, MODY10′s clinical spectrum—from neonatal diabetes to adult-onset hyperglycemia—is dictated by mutation-specific disruptions in proinsulin folding, trafficking, and ER homeostasis.

## 4. Molecular Mechanism of MODY10

Mutations in various regions of the *INS* gene can lead to different effects on protein synthesis, causing defective proinsulin folding, ER stress, β-cell apoptosis, and impaired insulin secretion.

**Figure 3:**
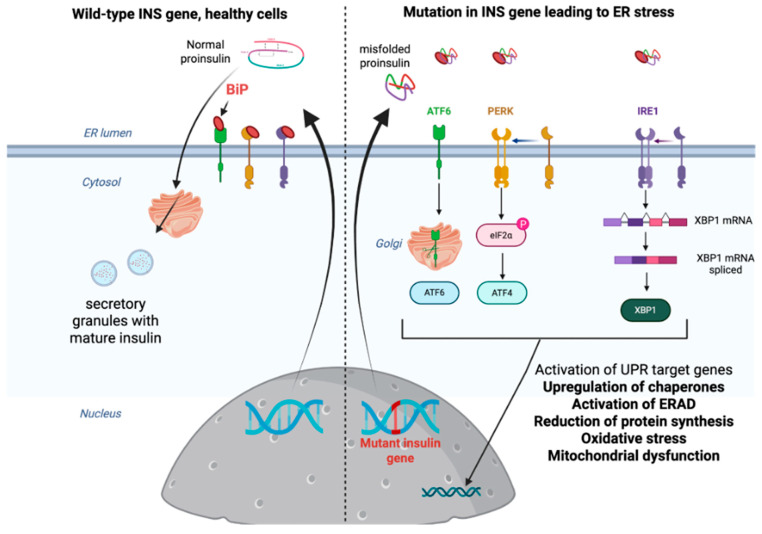
ER stress and unfolded protein response (UPR) activation in β-cells: Wild-type vs. *INS* mutant (MODY10) pathways. Created in Biorender.

Defective proinsulin folding in MODY10 results in the disruption of proinsulin’s structural integrity and processing, leading to the inability to synthesize mature insulin. Missense single nucleotide polymorphisms (SNPs) in the *INS* gene (mentioned earlier in Section 3), such as C43G, R6C, and G32S, can impair the disulfide bond formation (e.g., Cys43-Cys50 in the A-chain). At the same time, mutations altering the proteolytic cleavage sites between the B-chain and the C-chain (e.g., Arg6 at the B-chain N-terminus), can cause misfolded proinsulin that accumulates in the endoplasmic reticulum (ER) [19,20]. This misfolding triggers ER stress, activating the unfolded protein response (UPR), which includes three pathways: Protein kinase RNA-like ER kinase (PERK), Inositol-requiring enzyme 1 (IRE1), and Activating transcription factor 6 (ATF6). Ultimately, this promotes β-cell apoptosis through CHOP-mediated signaling (Figure 3) [21].

This figure illustrates the contrasting pathways of proinsulin processing in healthy β-cells and the endoplasmic reticulum (ER) stress response induced by mutations in the *INS* gene, as seen in MODY10. In wild-type cells (left panel), normal proinsulin folding occurs in the ER lumen with chaperone assistance (e.g., BiP), followed by efficient packaging into secretory granules for mature insulin secretion. In contrast, *INS* mutations (right panel) lead to misfolded proinsulin accumulation, triggering the unfolded protein response (UPR) through activation of ER stress sensors PERK (which phosphorylates eIF2α to attenuate translation), IRE1 (which splices XBP1 mRNA to upregulate folding chaperones and ER-associated degradation), and ATF6 (which translocate to the Golgi to induce stress-response genes). Chronic UPR activation shifts from an adaptive phase (chaperone upregulation, ERAD activation) to a pathological state characterized by oxidative stress, mitochondrial dysfunction, and ultimately β-cell apoptosis via CHOP and JNK signaling, highlighting the mechanistic link between *INS* mutations, ER stress, and insulin deficiency in MODY10. The figure underscores the dual role of UPR in maintaining proteostasis and driving β-cell failure under persistent stress; BiP—Binding Immunoglobulin Protein (also known as GRP78 or HSPA5); eIF2α—eukaryotic translation initiation factor 2; XBP1—X-box binding protein.

IRE1α, PERK, and ATF6 are all ER transmembrane proteins. The ER-luminal domain of IRE1α is regulated by the binding of BiP to it. Under ER stress conditions, BiP is released, allowing IRE1α to dimerize, transphosphorylate, and activate its endoribonuclease activity. This modifies the XBP1 coding mRNA by removing the intron and initiating the translation of XBP1 protein that further induces the expression of target genes that encode proteins with functions in ER protein folding, protein translocation into the ER, trafficking, and ER-associated degradation [22,23,24]. Simultaneously, PERK activation through dimerization leads to the phosphorylation of the α-subunit of eIF2α, resulting in the rapid and transient attenuation of general mRNA translation [24,25,26,27,28]. Paradoxically, the translation of several specific mRNAs is preferentially enhanced in the context of general translational inhibition, including ATF4, which plays an important role in antioxidant response and recovery of protein synthesis [29,30]. Finally, once released from the BiP, ATF6 translocates from the ER to the Golgi complex, where it is cleaved by sequential action of the membrane-bound transcription factor site 1 and site 2 proteases. This action liberates an ATF6 p50 cytosolic fragment from the N terminus of ATF6 that acts as a potent transcription factor to induce its target genes [31].

As mentioned by Liu et al., chronic PERK activation in β-cells promotes apoptosis via CHOP [19]. The IRE1α-XBP1s initially attempts to restore folding capacity but fails with mutant proinsulin accumulation, while ATF6 translocates to the Golgi when ER stress occurs and upregulates chaperone production, but this is insufficient to rescue misfolded proinsulin.

Additionally, mutant proinsulin exerts a dominant negative effect by competitively inhibiting prohormone convertases (PC1/3 and PC2), further reducing the cleavage of wild-type proinsulin and exacerbating insulin deficiency [32]. The resulting chronic ER stress and impaired insulin secretion contribute to the clinical manifestations of MODY10, which range from neonatal diabetes (severe mutations like R6C) to adult-onset hyperglycemia (milder variants like G32S) [33].

## 5. Current Management and Future Genetic Therapies for MODY10

### 5.1. Current Clinical Management

The current management of MODY10 focuses on addressing insulin deficiency while acknowledging the lack of therapies targeting the underlying genetic defect. Initial treatment typically involves sulfonylureas (e.g., glibenclamide) to stimulate residual insulin secretion from β-cells with preserved function, as some patients retain partial responsiveness due to the autosomal dominant nature of *INS* mutations. For those patients, sulfonylureas achieve partial glycemic control in ~30–50%, typically reducing HbA1c by 0.5–1.5% [34]. However, many cases progress to insulin dependence, particularly those with severe mutations (e.g., R6C), necessitating insulin therapy within 5–10 years of diagnosis to maintain glycemic control, with only 20% of patients maintaining sulfonylurea responsiveness long-term [35]. Continuous glucose monitoring (CGM) and personalized dosing regimens are employed to mitigate hypoglycemia risks, given the non-autoimmune etiology and variable insulin requirements. Genetic counseling is critical due to the 50% inheritance risk, and family screening is recommended to identify asymptomatic carriers.

### 5.2. AAV-Based Gene Therapy Approaches

A critical safety consideration in insulin gene therapy is preventing potentially dangerous insulin overexpression, which could lead to life-threatening hypoglycemia. This challenge requires precise regulatory systems in therapeutic approaches involving exogenous insulin expression. The risk of uncontrolled hyperinsulinemia remains one of the most significant barriers to developing safe and effective gene therapies for diabetes. Chen et al., mitigated hypoglycemia risk by designing a glucose-responsive system using the G6Pase promoter, which automatically adjusted insulin production based on blood glucose levels [36].

Earlier, Mas et al., who first established the concept of a “glucose sensor” in mice, co-expressed insulin and glucokinase genes in muscle cells simultaneously, leading to the regulation of blood glucose level [37]. Building upon this research, Jaén et al., and Callejas et al., stand out in their work focusing on long-term diabetes management. They demonstrated that a single administration of AAV1 co-expressing insulin and glucokinase in skeletal cells could maintain dogs (*n* = 6) with diabetes in a normoglycemic state for over 8 years without hypoglycemic episodes (HbA1c ≤ 6.5%), while also preventing secondary complications [38,39].

Other researchers have focused on alternative delivery approaches that can provide higher levels of transduction of target cells by AAV. For example, Park et al. (2005) used an AAV8-mediated portal vein delivery system, which successfully achieved stable hepatic insulin production in diabetic rats for over 90 days while also avoiding the hepatotoxicity associated with adenoviral vectors [40].

Gan et al., made a crucial advancement by engineering a liver-specific Tet-Off AAV8 vector system containing a codon-optimized human insulin gene. This system allowed precise pharmacological control of insulin secretion through doxycycline administration in diabetic mice [41]. Building on this concept of regulated expression, Qiao et al. (2024) developed an even more precise optogenetic system called red light-inducible photoswitch (REDLIP) that uses red/far-red light to control insulin production, representing a major leap forward in non-invasive, dynamic glycemic regulation [42]. However, Ramzy et al., identified an important limitation in insulin-deficient models. Insulin-deficient β-cells, such as those in MODY10, exhibit defective proinsulin processing, even after AAV-mediated insulin gene rescue. This suggests that simply restoring insulin expression may not be sufficient. Additional interventions to correct prohormone convertase (PC1/3) dysfunction or enhance secretory pathway efficiency may be required [43].

A potential complementary strategy was performed by Hashimoto et al., through cellular reprogramming approaches based on the *PDX-1* gene expression in hepatocytes. This process converted them into glucose-responsive insulin-secreting cells while still maintaining hepatic function [44]. Together, these studies demonstrate that AAV-based therapies can achieve durable, regulated insulin expression through multiple tissue targets and regulatory mechanisms, while also highlighting critical challenges in prohormone processing and long-term safety that must be addressed as these approaches move toward clinical application for monogenic forms of diabetes like MODY10. The diversity of successful strategies developed to date suggests that personalized approaches targeting different tissues and employing different regulatory mechanisms may be necessary to optimally treat the various forms of diabetes.

### 5.3. Non-AAV Therapeutic Strategies

He et al., demonstrated that non-viral hydrodynamic gene delivery can effectively transfer insulin-expressing plasmids to the livers of diabetic mice. This results in therapeutic insulin production that normalizes blood glucose levels and improves metabolic parameters. This approach, which utilizes rapid tail vein injection of naked DNA under a CMV promoter, successfully restored hepatic insulin expression. Despite limitations such as transient expression and scalability challenges, the results establish proof-of-concept for non-viral gene therapy in diabetes and suggest that further development of plasmid-based systems or integration of transposon technology could enhance its clinical potential [45].

Alternative gene therapy approaches have also shown promise for MODY10 treatment. Dr. Lu Deng et al., developed a novel plasmid-based gene therapy system (L/E-pSP301-SIA) that enables sustained expression of a single-stranded insulin analog (SIA) in skeletal muscle. This system replaced the rigid C-peptide in proinsulin with a short flexible linker and utilized a muscle-specific synthetic promoter (SP301) combined with Pluronic L64 and low-voltage electropulse delivery. The approach provided safe and effective long-term glucose control in diabetic mice for up to 1.5 months after a single intramuscular injection without triggering immune responses [46].

Emerging experimental approaches also include chemical chaperones (e.g., 4-PBA) to reduce ER stress caused by misfolded proinsulin, though these remain preclinical [47]. The efficacy of various gene therapy and ER stress-reduction approaches in preclinical diabetes models is summarized in Table 2.

These three presented systems are in the early stage of validation and still need modifications to increase efficacy. But they make a huge step in avoiding problems observed with AAV-based therapy, like immune response to the virus or tropism of the serotype.

### 5.4. Cell-Based Therapeutic Strategies for MODY10

#### 5.4.1. Islet Transplantation: Current Clinical Paradigm

Allogeneic islet transplantation, based on the Edmonton protocol, remains the only FDA-approved cell therapy for diabetes, offering MODY10 patients sustained glycemic control and reduced hypoglycemic episodes [49]. Despite its clinical validation, this approach faces significant limitations. The severe shortage of donor pancreases (with less than 3000 transplants performed globally since 1999) fails to meet the needs of over 1 million potential patients. Additionally, recipients require lifelong immunosuppression with calcineurin inhibitors (e.g., tacrolimus), which carry risks of nephrotoxicity and increased susceptibility to infection. Long-term studies reveal declining efficacy, with only 50% of grafts maintaining function at 5 years post-transplantation due to chronic immune rejection and β-cell apoptosis [50]. Recent refinements in islet isolation techniques and peritransplant anti-inflammatory regimens (e.g., TNF-α blockade) have marginally improved outcomes, but fundamental challenges persist.

#### 5.4.2. Stem Cell-Derived β-Cell Replacement

Pluripotent stem cell (PSC) technologies have emerged as a transformative solution to donor shortages. The VX-880 trial by Vertex Pharmaceuticals (NCT04786262) represents a landmark achievement, with fully differentiated pancreatic endoderm cells derived from human embryonic stem cells (ESCs) restoring endogenous insulin production in all 12 participants. Notably, 10 patients achieved insulin independence (HbA1c <7%) at the 1-year follow-up, with histopathology confirming mature islet structures containing insulin+ β-cells, glucagon+ α-cells, and somatostatin+ δ-cells [51]. Alternative approaches using induced pluripotent stem cells (iPSCs) circumvent ethical concerns but face reprogramming inconsistencies. Recent CRISPR-based epigenetic silencing of differentiation inhibitors (e.g., MEIS1) has improved protocol efficiency to over 80% β-cell purity [52,53,54]. Current differentiation protocols precisely mimic pancreatic development through sequential modulation of Wnt, Notch, and TGF-β signaling pathways, generating glucose-responsive islet clusters with dynamic insulin secretion profiles comparable to primary islets.

#### 5.4.3. Encapsulation Strategies for Immune Protection

Cell encapsulation technologies aim to physically shield transplanted islets from immune attack while permitting nutrient exchange. ViaCyte’s first-generation PEC-01 device employed macro-encapsulation of ESC-derived pancreatic progenitors in a semipermeable polytetrafluoroethylene (PTFE) membrane. Although well tolerated in clinical trials (NCT02239354), post-explant analysis revealed inadequate vascularization, with less than 10% of cells surviving beyond 6 months due to hypoxic necrosis [55,56,57]. Second-generation systems, such as Sernova’s Cell Pouch, address this through prevascularization surgical implantation of an empty scaffold 6–8 weeks prior to cell loading, which allows formation of a vascularized tissue matrix. This approach has demonstrated remarkable durability, with patients maintaining more than 5 years of islet function and insulin independence in ongoing trials (NCT03513939) [58]. Material science innovations continue to refine encapsulation, including:

- Alginate modifications: Covalent conjugation of RGD peptides to enhance cell-matrix adhesion
- Nanostructured membranes: Electrospun polycaprolactone fibers with tunable pore sizes (50–100 nm) for optimal immune isolation [55,59,60,61,62].

#### 5.4.4. Emerging Combinatorial Approaches

The integration of stem cell biology with immunomodulation represents the next frontier. Mesenchymal stem cell (MSC) co-transplantation leverages their paracrine effects, secreting VEGF to promote graft vascularization and IL-10/TGF-β to suppress T-cell responses. Clinical trials demonstrate that umbilical cord-derived MSCs reduce islet requirements by 40% while improving 5-year graft survival (62% vs. 29% in controls) [63,64,65,66,67,68]. Parallel advances in gene editing enable the creation of hypo-immune cells; CRISPR-engineered CTX-211 (NCT05565248) combines triple edits:

1. MHC-I knockout to prevent CD8+ T-cell recognition
2. PD-L1/HLA-E overexpression to inhibit NK cell activity
3. TNFAIP3/MANF co-expression to enhance stress resistance

Preclinical studies show these edits confer 75% protection against allorejection without immunosuppression [69,70,71,72]. Direct β-cell engineering has also identified protective mutations; RNLS knockout (associated with T1D GWAS loci) renders β-cells resistant to cytokine-induced apoptosis by upregulating NRF2 antioxidant pathways [69]. Current progress in clinical trials for beta-cell replacement therapies, ranging from allogeneic transplants to stem cell-derived and engineered cells, is detailed in Table 3.

### 5.5. Comparative Analysis and Future Perspective in MODY10 Therapeutics

Recent advances in MODY10 therapeutics present both opportunities and challenges when critically comparing emerging approaches. AAV-based gene therapies, such as those developed by Jaén et al. (2017) and Callejas et al. (2013), demonstrate remarkable durability, maintaining normoglycemia for over 8 years in canine models through hepatic insulin expression [38,39]. However, Ramzy et al. (2022) revealed a critical limitation: AAV-mediated insulin delivery fails to correct underlying prohormone convertase dysfunction in β-cells, suggesting viral vectors alone may be insufficient [43]. In contrast, non-viral approaches like Deng et al.’s (2023) plasmid-based single-chain insulin analog show shorter efficacy (6–8 weeks in mice) but avoid immunogenicity risks inherent to viral delivery [46]. For cell-based therapies, the Vertex VX-880 trial highlights the potential of stem cell-derived β-cells to restore physiological regulation, though scalability and cost remain substantial barriers compared to gene therapies. Notably, neither strategy currently addresses the dominant-negative effects of mutant proinsulin—a shared limitation with existing pharmacotherapies like sulfonylureas [34].

From our perspective, three priorities demand attention: First, direct comparative studies are needed to evaluate hepatic insulin secretion (AAV) versus engineered β-cell replacement, particularly regarding long-term safety and metabolic precision. Second, the persistent observation of mutant proinsulin aggregates [18] implies combinatorial strategies—perhaps pairing gene therapy with ER stress modulators like 4-PBA [47]—may be essential. Third, diagnostic pathways require optimization; more than 70% of MODY10 cases are misdiagnosed initially as T1D/T2D, delaying targeted interventions [74]. Future research should prioritize humanized models to bridge translational gaps, particularly for assessing dominant-negative inhibition and immune responses to AAV [41]. While no approach yet offers a complete solution, the convergence of gene editing, stem cell biology, and targeted protein correction holds transformative potential for this monogenic diabetes subtype.

## 6. Conclusions

MODY10 represents a compelling model of monogenic diabetes, where *INS* mutations disrupt insulin biosynthesis through defective proinsulin processing and ER stress-mediated β-cell dysfunction. Current management uses sulfonylureas or insulin, but emerging AAV-based and plasmid-delivered gene therapies show promise for addressing the underlying genetic defect. Preclinical studies demonstrate that “glucose-responsive” insulin expression systems can achieve sustained glycemic control while avoiding hypoglycemia, highlighting their therapeutic potential. These advances, coupled with improved genetic diagnostics, pave the way for precision medicine approaches to MODY10 treatment. Cell-based therapies for MODY10, including islet transplantation and stem cell-derived β-cell replacement, offer promising solutions for restoring glycemic control but face challenges such as donor shortages, immune rejection, and the need for lifelong immunosuppression. Emerging technologies like encapsulation strategies and CRISPR-engineered hypo-immune cells aim to overcome these limitations, paving the way for more durable and accessible treatments.

## 7. Limitations

Despite significant progress in characterizing MODY10, critical knowledge gaps remain. The incomplete understanding of genotype-phenotype variability—particularly why some mutation carriers remain normoglycemic—suggests undiscovered genetic or environmental modifiers. The precise mechanisms by which mutant proinsulin exerts dominant-negative effects and whether its aggregates are independently cytotoxic remain unclear. Therapeutic challenges persist, including whether gene therapy can fully overcome mutant proinsulin’s effects and the long-term safety of sustained hepatic insulin expression. Clinically, standardized criteria for genetic testing and data on comorbidities (e.g., cardiovascular risk) are lacking. Future research should prioritize longitudinal natural history studies, humanized models, and screens for ER stress mitigators to address these gaps.

Moreover, existing studies are primarily limited to rodent models, which may not fully recapitulate human pathophysiology or long-term safety concerns. Despite the promising results from preclinical studies, several critical limitations must be addressed before AAV-mediated gene therapy can be widely applied for diabetes treatment. These limitations include immune responses and vector persistence, prohormone processing defects, risk of hypoglycemia and lack of physiological regulation, tissue-specific challenges (AAV8 and risk of hepatotoxicity, AAV1 potentially leading to lower insulin secretion in muscle), dose-dependent toxicity and off-target effects, as well as translational barriers in large animals and humans (such as different immunity and AAV serotype tropism).

Finally, current cell-based therapies for MODY10 remain constrained by donor scarcity, inconsistent graft survival, and reliance on immunosuppression, while stem cell-derived solutions face challenges in scalability, functional maturation, and long-term safety. Additionally, encapsulation technologies struggle with suboptimal vascularization and immune protection, highlighting the need for further innovation in biocompatibility and precision immune modulation.

## Figures and Tables

**Table 1:** Confirmed MODY subtypes [6,11].

MODY Type	Gene	Chromosome	Key Features	Treatment
MODY1	*HNF4Alpha*	20q13.12	Neonatal hypoglycemia, macrosomia; progressive insulin deficiency	Sulfonylureas (effective), may need insulin later
MODY2	*GCK*	7p13	Mild, stable fasting hyperglycemia; rarely requires treatment	Usually no therapy; insulin if misdiagnosed
MODY3	*HNF1A*	12q24.31	Progressive β-cell dysfunction; renal glycosuria	Sulfonylureas (1st line), insulin eventually
MODY4	*PDX1*	13q12.2	Pancreatic agenesis (severe forms); mild diabetes in heterozygotes	Insulin (if deficient), some respond to sulfonylureas
MODY5	*HNF1B*	17q12	Renal cysts, genital malformations, gout; diabetes often secondary	Insulin (due to multi-organ involvement)
MODY6	*NEUROD1*	2q31.3	Rare; neurological features in some cases	Insulin or sulfonylureas (variable)
MODY7	*KLF11*	2p25.1	Extremely rare; mild hyperglycemia	Diet/lifestyle, rarely medications
MODY8	*CEL*	9q34.13	Exocrine pancreas dysfunction, fatty stools	Insulin (pancreatic insufficiency)
MODY9	*PAX4*	7q32.1	Reported in Asian populations; ketoacidosis risk	Insulin typically required
MODY10	*INS*	11p15.5	Defective insulin processing; non-autoimmune	Sulfonylureas (if residual function), else insulin
MODY11	*BLK*	8p23.1	Mild diabetes; reduced β-cell mass	Variable (diet to insulin)
MODY12	*ABCC8*	11p15.1	Neonatal diabetes overlap; may respond to sulfonylureas	High-dose sulfonylureas (often effective)
MODY13	*KCNJ11*	11p15.1	Neonatal diabetes spectrum; ATP-sensitive K+ channel defect	Sulfonylureas (90% respond)
MODY14	*APPL1*	3p14.3	Recent discovery; insulin secretion defect	Insufficient data

**Table 2:** Comparative analysis of non-viral gene therapy strategies.

Approach	Delivery Method	Study Subject	Duration	Efficacy Data	Citations
Hydrodynamic plasmid	High-pressure tail vein	Diabetic mice (STZ-induced)	In vivo	Normalized blood glucose for 2–4 weeks; transient expression.	[45]
L/E-pSP301-SIA plasmid	Electropulse + Pluronic	STZ-induced diabetic mice (C57BL/6)	In vivo	Sustained normoglycemia for 6–8 weeks post-intramuscular delivery.	[46]
Chemical chaperones (4-PBA)	Systemic administration	Human iPSC-derived β-cells (WFS1 mutations)	In vitro	4-PBA reduced ER stress markers (BiP, CHOP) by 50% and restored glucose-stimulated insulin secretion (GSIS) by 30%. Demonstrated cross-relevance to monogenic diabetes ER stress pathways.	[47,48]

**Table 3:** Cell therapy clinical pipeline for MODY10.

Strategy	Study Subject	Representative Trial	Phase	Efficacy Data	Citations
Allogeneic islets	T1D patients (*n* = 1210)	Collaborative Islet Transplant Registry (CITR)	Phase 3	75% insulin independence at 5 years in PGF+ patients (median HbA1c 5.6% in responders)	[49,50,69,73]
ESC-derived β-cells	T1D patients (*n* = 17)	Vertex VX-880 (NCT04786262)	Phase I/II	71% insulin independence at 1 year (HbA1c ≤ 7% in 10/12 patients)	[51]
Encapsulation device	T1D patients (ongoing trial)	Sernova Cell Pouch (NCT03513939)	Phase I/II	5+ years sustained function in ongoing trial	[58] [NCT03513939]
Gene-edited hypo-immune	Humanized mice (NSG model *n* = 10)	CTX-211 (NCT05565248)	Phase I/II	90% rejection protection (preclinical)	[69,70,71,72] [NCT05565248]

Footnotes: PGF+: Primary Graft Function. Phase I/II: Early-stage trials testing safety (Phase I) and preliminary efficacy (Phase II) in small patient groups. Phase 3: Large-scale trials confirming efficacy, monitoring side-effects, and comparing to standard treatments.

## References

[1] ElSayed N.A., Aleppo G., Aroda V.R., Bannuru R.R., Brown F.M., Bruemmer D., Collins B.S., Gaglia J.L., Hilliard M.E., Isaacs D. (2022). 2. Classification and Diagnosis of Diabetes: Standards of Care in Diabetes—2023. Diabetes Care.

[2] International Diabetes Federation (2024). The Diabetes Atlas.

[3] Atkinson M.A., Eisenbarth G.S., Michels A.W. (2014). Type 1 diabetes. Lancet.

[4] DiMeglio L.A., Evans-Molina C., Oram R.A. (2018). Type 1 diabetes. Lancet.

[5] Rohm T.V., Meier D.T., Olefsky J.M., Donath M.Y. (2022). Inflammation in obesity, diabetes, and related disorders. Immunity.

[6] McDonald T.J., McEneny J., Pearson E.R., Thanabalasingham G., Szopa M., Shields B.M., Ellard S., Owen K.R., Malecki M.T., Hattersley A.T. (2012). Lipoprotein composition in HNF1A-MODY: Differentiating between HNF1A-MODY and Type 2 diabetes. Clin. Chim. Acta.

[7] Tattersall R.B., Fajans S.S., Arbor A. (1975). A Difference Between the Inheritance of Classical Juvenile-onset and Maturity-onset Type Diabetes of Young People. Diabetes.

[8] Shields B.M., Hicks S., Shepherd M.H., Colclough K., Hattersley A.T., Ellard S. (2010). Maturity-onset diabetes of the young (MODY): How many cases are we missing?. Diabetologia.

[9] Misra S., Owen K.R. (2018). Genetics of Monogenic Diabetes: Present Clinical Challenges. Curr. Diabetes Rep..

[10] Yang Y., Chan L. (2016). Monogenic Diabetes: What it teaches us on the common forms of type 1 and type 2 diabetes. Endocr. Rev. Endocr. Soc..

[11] Oliveira S.C., Neves J.S., Pérez A., Carvalho D. (2020). Maturity-onset diabetes of the young: From a molecular basis perspective toward the clinical phenotype and proper management. Endocrinol. Diabetes Nutr..

[12] Ataie-Ashtiani S., Forbes B. (2023). A Review of the Biosynthesis and Structural Implications of Insulin Gene Mutations Linked to Human Disease. Cells.

[13] Guo H., Xiong Y., Witkowski P., Cui J., Wang L.-J., Sun J., Lara-Lemus R., Haataja L., Hutchison K., Shan S.-O. (2014). Inefficient translocation of preproinsulin contributes to pancreatic β cell failure and late-onset diabetes. J. Biol. Chem..

[14] Liu M., Lara-Lemus R., Shan S.-O., Wright J., Haataja L., Barbetti F., Guo H., Larkin D., Arvan P. (2012). Impaired cleavage of preproinsulin signal peptide linked to autosomal-dominant diabetes. Diabetes.

[15] Zhu R., Li X., Xu J., Barrabi C., Kekulandara D., Woods J., Chen X., Liu M. (2019). Defective endoplasmic reticulum export causes proinsulin misfolding in pancreatic β cells. Mol. Cell. Endocrinol..

[16] Jang I., Pottekat A., Poothong J., Yong J., Lagunas-Acosta J., Charbono A., Chen Z., Scheuner D.L., Liu M., Itkin-Ansari P. (2019). PDIA1/P4HB is required for efficient proinsulin maturation and ß cell health in response to diet induced obesity. eLife.

[17] Zito E., Chin K.T., Blais J., Harding H.P., Ron D. (2010). ERO1-beta, a pancreas-specific disulfide oxidase, promotes insulin biogenesis and glucose homeostasis. J. Cell Biol..

[18] Wang H., Saint-Martin C., Xu J., Ding L., Wang R., Feng W., Liu M., Shu H., Fan Z., Haataja L. (2020). Biological behaviors of mutant proinsulin contribute to the phenotypic spectrum of diabetes associated with insulin gene mutations. Mol. Cell. Endocrinol..

[19] Liu M., Huang Y., Xu X., Li X., Alam M., Arunagiri A., Haataja L., Ding L., Wang S., Itkin-Ansari P. (2021). Normal and defective pathways in biogenesis and maintenance of the insulin storage pool. J. Clin. Investig..

[20] Støy J., Edghill E.L., Flanagan S.E., Ye H., Paz V.P., Pluzhnikov A., Below J.E., Hayes M.G., Cox N.J., Lipkind G.M. (2007). Insulin gene mutations as a cause of permanent neonatal diabetes. Proc. Natl. Acad. Sci. USA.

[21] Wang Y.-C., Li X., Shen Y., Lyu J., Sheng H., Paschen W., Yang W. (2020). PERK (Protein Kinase RNA-Like ER Kinase) Branch of the Unfolded Protein Response Confers Neuroprotection in Ischemic Stroke by Suppressing Protein Synthesis. Stroke.

[22] Yong J., Johnson J.D., Arvan P., Han J., Kaufman R.J. (2021). Therapeutic opportunities for pancreatic β-cell ER stress in diabetes mellitus. Nat. Rev. Endocrinol..

[23] Shen X., Ellis R., Sakaki K., Kaufman R.J., Kim S. (2005). Genetic Interactions Due to Constitutive and Inducible Gene Regulation Mediated by the Unfolded Protein Response in C. elegans. PLoS Genet..

[24] Hassler J.R., Scheuner D.L., Wang S., Han J., Kodali V.K., Li P., Nguyen J., George J.S., Davis C., Wu S.P. (2015). The IRE1α/XBP1s Pathway Is Essential for the Glucose Response and Protection of β Cells. PLoS Biol..

[25] Prostko C., Brostrom M., Malara E., Brostrom C. (1992). Phosphorylation of eukaryotic initiation factor (eIF) 2 alpha and inhibition of eIF-2B in GH3 pituitary cells by perturbants of early protein processing that induce GRP78. J. Biol. Chem..

[26] Harding H.P., Zhang Y., Ron D. (1999). Protein translation and folding are coupled by an endoplasmic-reticulum-resident kinase. Nature.

[27] Scheuner D., Song B., McEwen E., Liu C., Laybutt R., Gillespie P., Saunders T., Bonner-Weir S., Kaufman R.J. (2001). Translational Control Is Required for the Unfolded Protein Response and In Vivo Glucose Homeostasis. Mol. Cell.

[28] Yong J., Grankvist N., Han J., Kaufman R.J. (2014). Eukaryotic translation initiation factor 2 α phosphorylation as a therapeutic target in diabetes. Expert Rev. Endocrinol. Metab..

[29] Harding H.P., Novoa I., Zhang Y., Zeng H., Wek R., Schapira M., Ron D. (2000). Regulated Translation Initiation Controls Stress-Induced Gene Expression in Mammalian Cells. Mol. Cell.

[30] Han J., Back S.H., Hur J., Lin Y.-H., Gildersleeve R., Shan J., Yuan C.L., Krokowski D., Wang S., Hatzoglou M. (2013). ER-stress-induced transcriptional regulation increases protein synthesis leading to cell death. Nat. Cell Biol..

[31] Haze K., Yoshida H., Yanagi H., Yura T., Mori K., Silver P. (1999). Mammalian Transcription Factor ATF6 Is Synthesized as a Transmembrane Protein and Activated by Proteolysis in Response to Endoplasmic Reticulum Stress. Mol. Biol. Cell.

[32] Zhu X., Orci L., Carroll R., Norrbom C., Ravazzola M., Steiner D.F. (2002). Severe block in processing of proinsulin to insulin accompanied by elevation of des-64,65 proinsulin intermediates in islets of mice lacking prohormone convertase 1/3. Proc. Natl. Acad. Sci. USA.

[33] Polak M., Dechaume A., Cavé H., Nimri R., Crosnier H., Sulmont V., de Kerdanet M., Scharfmann R., Lebenthal Y., Froguel P. (2008). Heterozygous Missense Mutations in the Insulin Gene Are Linked to Permanent Diabetes Appearing in the Neonatal Period or in Early Infancy: A Report From the French ND (Neonatal Diabetes) Study Group. Diabetes.

[34] Pearson J., Powers M.A. (2006). Systematically Initiating Insulin. Diabetes Educ..

[35] Murphy R., Ellard S., Hattersley A.T. (2008). Clinical implications of a molecular genetic classification of monogenic β-cell diabetes. Nat. Clin. Pract. Endocrinol. Metab..

[36] Chen R., Meseck M.L., Woo S.L. (2001). Auto-regulated hepatic insulin gene expression in type 1 diabetic rats. Mol. Ther..

[37] Mas A., Montané J., Anguela X.M., MuñoZ S., Douar A.M., Riu E., Otaegui P., Bosch F. (2006). Reversal of type 1 diabetes by engineering a glucose sensor in skeletal muscle. Diabetes.

[38] Jaén M.L., Vilà L., Elias I., Jimenez V., Rodó J., Maggioni L., Gopegui R.R.-D., Garcia M., Muñoz S., Callejas D. (2017). Long-Term Efficacy and Safety of Insulin and Glucokinase Gene Therapy for Diabetes: 8-Year Follow-Up in Dogs. Mol. Ther.-Methods Clin. Dev..

[39] Callejas D., Mann C.J., Ayuso E., Lage R., Grifoll I., Roca C., Andaluz A., Gopegui R.R.-D., Montané J., Muñoz S. (2013). Treatment of Diabetes and Long-Term Survival After Insulin and Glucokinase Gene Therapy. Diabetes.

[40] Park Y.M., Woo S., Lee G.T., Ko J., Lee Y., Zhao Z., Kim H.J., Ahn C.W., Cha B.S., Kim K. (2005). Safety and efficacy of adeno-associated viral vector-mediated insulin gene transfer via portal vein to the livers of streptozotocin-induced diabetic Sprague-Dawley rats. J. Gene Med..

[41] Gan S.U., Fu Z., Sia K.C., Kon O.L., Calne R., Lee K.O. (2019). Development of a liver-specific Tet-off AAV8 vector for improved safety of insulin gene therapy for diabetes. J. Gene Med..

[42] Qiao L., Niu L., Wang M., Wang Z., Kong D., Yu G., Ye H. (2024). A sensitive red/far-red photoswitch for controllable gene therapy in mouse models of metabolic diseases. Nat. Commun..

[43] Ramzy A., Edeer N., Baker R.K., O’dWyer S., Mojibian M., Verchere C.B., Kieffer T.J. (2022). Insulin Null β-cells Have a Prohormone Processing Defect That Is Not Reversed by AAV Rescue of Proinsulin Expression. Endocrinology.

[44] Hashimoto H., Higuchi Y., Kawai K. (2015). Forced expression of PDX-1 gene makes hepatoma cells to acquire glucose-responsive insulin secretion while maintaining hepatic characteristic. Cell. Mol. Biol..

[45] He C.X., Shi D., Wu W.J., Ding Y.F., Feng D.M., Lu B., Chen H.M., Yao J.-H., Shen Q., Lu D.-R. (2004). Insulin expression in livers of diabetic mice mediated by hydrodynamics-based administration. World J. Gastroenterol..

[46] Deng L., Yang P., Li C., Xie L., Lu W., Zhang Y., Liu M., Wang G. (2023). Prolonged control of insulin-dependent diabetes via intramuscular expression of plasmid-encoded single-strand insulin analogue. Genes Dis..

[47] Ghaderi S., Ahmadian S., Soheili Z.S., Ahmadieh H., Samiei S., Kheitan S., Pirmardan E.R. (2018). AAV delivery of GRP78/BiP promotes adaptation of human RPE cell to ER stress. J. Cell. Biochem..

[48] Shang L., Hua H., Foo K., Martinez H., Watanabe K., Zimmer M., Kahler D.J., Freeby M., Chung W., LeDuc C. (2014). β-cell dysfunction due to increased ER stress in a stem cell model of Wolfram syndrome. Diabetes.

[49] U.S. Food and Drug Administration (2009). Guidance for Industry: Considerations for Allogeneic Pancreatic Islet Cell Products. https://www.fda.gov/downloads/BiologicsBloodVaccines/GuidanceComplianceRegulatoryInformation/Guidances/CellularandGeneTherapy/UCM182441.pdf.

[50] Marfil-Garza B.A., Shapiro A.M.J., Kin T. (2021). Clinical islet transplantation: Current progress and new frontiers. J. Hepato-Biliary-Pancreat. Sci..

[51] Reichman T.W., Markmann J.F., Odorico J., Witkowski P., Fung J.J., Wijkstrom M., Kandeel F., de Koning E.J., Peters A.L., Mathieu C. (2025). Stem Cell–Derived, Fully Differentiated Islets for Type 1 Diabetes. N. Engl. J. Med..

[52] Zakrzewski W., Dobrzyński M., Szymonowicz M., Rybak Z. (2019). Stem cells: Past, present, and future. Stem Cell Res Ther..

[53] Pagliuca F.W., Millman J.R., Gürtler M., Segel M., Van Dervort A., Ryu J.H., Peterson Q.P., Greiner D., Melton D.A. (2014). Generation of Functional Human Pancreatic β Cells In Vitro. Cell.

[54] Chen J.T., Dadheech N., Tan E.H.P., Ng N.H.J., Koh M.B.C., Shapiro J., Teo A.K.K. (2025). Stem cell therapies for diabetes. Nat. Med..

[55] Ramzy A., Thompson D.M., Ward-Hartstonge K.A., Ivison S., Cook L., Garcia R.V., Loyal J., Kim P.T., Warnock G.L., Levings M.K. (2021). Implanted pluripotent stem-cell-derived pancreatic endoderm cells secrete glucose-responsive C-peptide in patients with type 1 diabetes. Cell Stem Cell.

[56] Rezania A., Bruin J.E., Riedel M.J., Mojibian M., Asadi A., Xu J., Gauvin R., Narayan K., Karanu F., O’nEil J.J. (2012). Maturation of Human Embryonic Stem Cell–Derived Pancreatic Progenitors Into Functional Islets Capable of Treating Pre-existing Diabetes in Mice. Diabetes.

[57] Kroon E., Martinson L.A., Kadoya K., Bang A.G., Kelly O.G., Eliazer S., Young H., Richardson M., Smart N.G., Cunningham J. (2008). Pancreatic endoderm derived from human embryonic stem cells generates glucose-responsive insulin-secreting cells in vivo. Nat. Biotechnol..

[58] Gazia C., Gaffley M., Asthana A., Chaimov D., Orlando G., Mozafari M., Sefat F., Atala A. (2019). 65-Scaffolds for pancreatic tissue engineering. Handbook of Tissue Engineering Scaffolds.

[59] Li H., He W., Feng Q., Chen J., Xu X., Lv C., Zhu C., Dong H. (2024). Engineering superstable islets-laden chitosan microgels with carboxymethyl cellulose coating for long-term blood glucose regulation in vivo. Carbohydr. Polym..

[60] Alagpulinsa D.A., Cao J.J., Driscoll R.K., Sîrbulescu R.F., Penson M.F., Sremac M., Engquist E.N., Brauns T.A., Markmann J.F., Melton D.A. (2019). Alginate-microencapsulation of human stem cell–derived β cells with CXCL12 prolongs their survival and function in immunocompetent mice without systemic immunosuppression. Am. J. Transplant..

[61] Stock A.A., Manzoli V., De Toni T., Abreu M.M., Poh Y.-C., Ye L., Roose A., Pagliuca F.W., Thanos C., Ricordi C. (2020). Conformal Coating of Stem Cell-Derived Islets for β Cell Replacement in Type 1 Diabetes. Stem Cell Rep..

[62] Liu S.S., Shim S., Kudo Y., Stabler C.L., O’Cearbhaill E.D., Karp J.M., Yang K. (2024). Encapsulated islet transplantation. Nat. Rev. Bioeng..

[63] Goodarzi P., Larijani B., Alavi-Moghadam S., Tayanloo-Beik A., Mohamadi-Jahani F., Ranjbaran N., Payab M., Falahzadeh K., Mousavi M., Arjmand B., Turksen K. (2018). Mesenchymal Stem Cells-Derived Exosomes for Wound Regeneration. Cell Biology and Translational Medicine, Volume 4: Stem Cells and Cell Based Strategies in Regeneration.

[64] Maacha S., Sidahmed H., Jacob S., Gentilcore G., Calzone R., Grivel J.-C., Cugno C. (2020). Paracrine Mechanisms of Mesenchymal Stromal Cells in Angiogenesis. Stem Cells Int..

[65] Schive S.W., Mirlashari M.R., Hasvold G., Wang M., Josefsen D., Gullestad H.P., Korsgren O., Foss A., Kvalheim G., Scholz H. (2017). Human Adipose-Derived Mesenchymal Stem Cells Respond to Short-Term Hypoxia by Secreting Factors Beneficial for Human Islets in Vitro and Potentiate Antidiabetic Effect in Vivo. Cell Med..

[66] Hubber E.L., Rackham C.L., Jones P.M. (2021). Protecting islet functional viability using mesenchymal stromal cells. Stem Cells Transl. Med..

[67] Hu J., Yu X., Wang Z., Wang F., Wang L., Gao H., Chen Y., Zhao W., Jia Z., Yan S. (2013). Long term effects of the implantation of Wharton’s jelly-derived mesenchymal stem cells from the umbilical cord for newly-onset type 1 diabetes mellitus. Endocr. J..

[68] Izadi M., Nejad A.S.H., Moazenchi M., Masoumi S., Rabbani A., Kompani F., Asl A.A.H., Kakroodi F.A., Jaroughi N., Meybodi M.A.M. (2022). Mesenchymal stem cell transplantation in newly diagnosed type-1 diabetes patients: A phase I/II randomized placebo-controlled clinical trial. Stem Cell Res. Ther..

[69] Cai E.P., Ishikawa Y., Zhang W., Leite N.C., Li J., Hou S., Kiaf B., Hollister-Lock J., Yilmaz N.K., Schiffer C.A. (2020). Genome-scale in vivo CRISPR screen identifies RNLS as a target for beta cell protection in type 1 diabetes. Nat. Metab..

[70] Lim D., Sreekanth V., Cox K.J., Law B.K., Wagner B.K., Karp J.M., Choudhary A. (2020). Engineering designer beta cells with a CRISPR-Cas9 conjugation platform. Nat. Commun..

[71] Gerace D., Zhou Q., Kenty J.H.-R., Veres A., Sintov E., Wang X., Boulanger K.R., Li H., Melton D.A. (2023). Engineering human stem cell-derived islets to evade immune rejection and promote localized immune tolerance. Cell Rep. Med..

[72] Yoshihara E., O’Connor C., Gasser E., Wei Z., Oh T.G., Tseng T.W., Wang D., Cayabyab F., Dai Y., Yu R.T. (2020). Immune-evasive human islet-like organoids ameliorate diabetes. Nature.

[73] Chetboun M., Drumez E., Ballou C., Maanaoui M., Payne E., Barton F., Kerr-Conte J., Vantyghem M.-C., Piemonti L., Rickels M.R. (2023). Association between primary graft function and 5-year outcomes of islet allogeneic transplantation in type 1 diabetes: A retrospective, multicentre, observational cohort study in 1210 patients from the Collaborative Islet Transplant Registry. Lancet Diabetes Endocrinol..

[74] Chen H., Fei S.-J., Deng M.-Q., Chen X.-D., Wang W.-H., Guo L.-X., Pan Q. (2023). Maturity-onset diabetes of the young type 10 caused by an Ala2Thr mutation of INS: A case report. World J. Diabetes.

